# Supplementary material for: New insight into the role of MMP14 in metabolic balance
Source: PeerJ. 2016 Jul 13;4:e2142. doi: 10.7717/peerj.2142 (PMC4950575; doi:10.7717/peerj.2142)
Supplement: Table S1 — TG = triglyceride/SM = sphingomyelin/PC = phosphatidylcholine/lysoPC = lysophosphatidylcholine/PI = phosphoinositol Note: Lipid notation (X:Y), in which X = total number of carbons and Y = total number of double bonds in lipid acyl chains Note: For diglyceride and TG lipids specified here, these represent isomers (identical m∕z and chemical formula) of that lipid species with the same total number of carbons and double bonds but with differing acyl chain lengths. [file peerj-04-2142-s003.docx]

| **Compound** | **Formula** | **Neutral Mass** | ***m/z* (detected)** | **adduct** | ***m/z* difference (ppm)** | **Retention time, peak (min)** |
| --- | --- | --- | --- | --- | --- | --- |
| lysoPC(16:0) | C24H50NO7P | 495.3325 | 496.3445 | H+ | 9.39 | 10.43 |
| lysoPC(18:0) | C26H54NO7P | 523.3638 | 524.3738 | H+ | 5.08 | 12.24 |
| lysoPC(18:2) | C26H50NO7P | 519.3325 | 520.3446 | H+ | 9.15 | 8.99 |
| lysoPC(18:2) | C26H50NO7P | 519.3325 | 520.3446 | H+ | 9.15 | 9.38 |
| PC(30:0) | C38H76NO8P | 705.5309 | 706.5421 | H+ | 5.52 | 22.00 |
| PC(34:1) | C42H82NO8P | 759.5778 | 760.5890 | H+ | 5.06 | 24.64 |
| PC(34:2) | C42H80NO8P | 757.5622 | 758.5671 | H+ | 3.17 | 22.69 |
| PC(O-32:0) | C40H84NO6P | 705.6036 | 706.6070 | H+ | 5.62 | 25.25 |
| PI(38:4) | C47H83O13P | 886.5571 | 904.5915 | NH4+ | 0.63 | 15.70 |
| SM(d18:0/16:1) | C39H79N2O6P | 702.5676 | 703.5782 | H+ | 4.66 | 19.68 |
| SM(d18:2/16:0) | C39H77N2O6P | 700.5519 | 701.5624 | H+ | 4.46 | 18.18 |
| TG(14:1/14:1/14:1) | C45H80O6 | 716.5955 | 734.6322 | NH4+ | 3.95 | 27.81 |
| TG(14:1/16:0/14:1) | C47H86O6 | 746.6424 | 764.6813 | NH4+ | 6.61 | 29.44 |
| TG(14:1/16:1/14:1) | C47H84O6 | 744.6268 | 762.6655 | NH4+ | 6.43 | 28.69 |
